# *ctdsp2* Knockout Induces Zebrafish Craniofacial Dysplasia via p53 Signaling Activation

**DOI:** 10.3390/ijms26031297

**Published:** 2025-02-03

**Authors:** Xin Xia, Wenjie Song, Fuyu Zhang, Yue Fan, Bo Zhang, Xiaowei Chen

**Affiliations:** 1 Department of Otolaryngology-Head and Neck Surgery, Peking Union Medical College Hospital, Peking Union Medical College and Chinese Academy of Medical Sciences, Beijing 100730, China; 2 Eight-Year MD Program, Peking Union Medical College and Chinese Academy of Medical Sciences, Beijing 100730, China; 3 Key Laboratory of Cell Proliferation and Differentiation of the Ministry of Education, College of Life Sciences, Peking University, Beijing 100871, China

**Keywords:** *ctdsp2*, zebrafish, craniofacial defects, neural crest cell, p53 signaling pathway

## Abstract

Hemifacial microsomia (HFM) is a rare congenital craniofacial deformity that significantly impacts the appearance and hearing. The genetic etiology of HFM remains largely unknown, although genetic factors are considered to be primary contributors. We previously identified *CTDSP2* as a potential causative gene in HFM cases. Utilizing CRISPR/Cas9, we knocked out *ctdsp2* in zebrafish and analyzed the spatiotemporal expression of *ctdsp2* and neural crest cell (NCC) markers through in situ hybridization (ISH). Craniofacial cartilage and chondrocyte phenotypes were visualized using Alcian blue and wheat germ agglutinin (WGA) staining. Cell proliferation and apoptosis were assessed via immunofluorescence with PH3 and TUNEL. RNA sequencing was performed on *ctdsp2*^−/−^ embryos and control siblings, followed by rescue experiments. Knockout of *ctdsp2* in zebrafish resulted in craniofacial defects characteristic of HFM. We observed abnormalities in NCC apoptosis and proliferation in the pharyngeal arches, as well as impaired differentiation of chondrocytes in *ctdsp2*^−/−^ embryos. RNA-Seq analysis revealed significantly higher expression of genes in the p53 signaling pathway in mutants. Furthermore, *ctdsp2* mRNA injection and *tp53* knockout significantly rescued pharyngeal arch cartilage dysplasia. Our findings suggest that *ctdsp2* knockout induces zebrafish craniofacial dysplasia, primarily by disrupting pharyngeal chondrocyte differentiation and inhibiting NCC proliferation through p53 signaling pathway activation.

## 1. Introduction

Hemifacial microsomia (HFM) is a congenital craniofacial disorder characterized by unilateral hypoplasia of facial structures, leading to facial asymmetry, ear malformations, and underdeveloped jawbones [1]. Although HFM is relatively infrequent, with an estimated incidence rate of 1:3500 to 1:4500 live births, it significantly affects a small yet critical demographic [2].

The etiology of HFM is complex and multifactorial, with genetic factors playing a major role [3]. Currently, several pathogenic genes and single-nucleotide variants have been proposed for HFM. In 2016, Lopez et al. reported the first pathogenic gene for HFM, *MYT1*, via whole-genome sequencing in two sporadic pedigrees [4]. Subsequent observations of its variants in other cohorts have considered it to be an important cause of HFM [5,6,7]. Moreover, *SF3B2* and *FOXI3* have been identified as causative genes from several cohorts, each explaining the etiology of approximately 1–3% of the probands [8,9,10]. In addition to these well-studied genes, several other genes are suggested to be associated with HFM, such as *ZYG11B*, *EYA3*, *FRK*, and *CDC27* [11,12,13,14], yet their pathogenicity is pending confirmation. Despite extensive research, the specific genes and mechanisms responsible for HFM have not been fully elucidated. In our quest to identify the genetic factors contributing to HFM, we conducted a trio whole-exome sequencing (trio-WES) study across 12 sporadic families [14]. We identified a de novo rare variant in *CTDSP2*, a gene not previously associated with HFM, in two unrelated cases. This variant, which is non-synonymous and predicted to be pathogenic, emerged as a potential cause of the disorder. *CTDSP2*, encoding a protein from the small CTD phosphatase family, is crucial for human cellular functions. It primarily regulates RNA polymerase II (RNAPII) activity by dephosphorylating its C-terminal domain (CTD). This activity plays a key role in the transcription process, and the dephosphorylation is essential for the proper progression and termination of transcription, which impacts mRNA synthesis and downstream cellular processes like growth, differentiation, and stress response [15]. Notably, aberrant expression or dysfunction of *CTDSP2* can give rise to abnormal gene expression associated with cell-cycle regulation, apoptosis, and angiogenesis [16]. However, its pathogenicity has not been verified in animal models, while its pathogenic mechanism remains unanswered.

To ascertain whether *CTDSP2* is a candidate gene for HFM, we generated a *ctdsp2*-knockout zebrafish model, which exhibited craniofacial defects characterized by cartilage malformations and chondrocyte disorganization. To elucidate the pathogenic mechanism of *CTDSP2*, we conducted a combination of in silico and in vivo experiments to assess the impact of *CTDSP2* on the development of neural crest cells (NCCs), a process pivotal to craniofacial development [17]. Bioinformatics analysis suggested potential interactions between *CTDSP2* and proteins essential for chondrogenesis and the TGF-β pathway. On the other hand, in *ctdsp2*^−/−^ larvae, in situ hybridization (ISH) and qRT-PCR demonstrated decreased expression of chondrogenic markers, while RNA-Seq analysis and rescue experiments highlighted the engagement of the p53 pathway in the *ctdsp2*-knockout phenotype. Collectively, our findings illuminate the genetic basis and pathogenic mechanisms underlying HFM, offering insights for the development of screening and therapeutic strategies for this disorder.

## 2. Results

### 2.1. Novel Variant Identification and Molecular Analysis of CTDSP2

**Figure 1:**
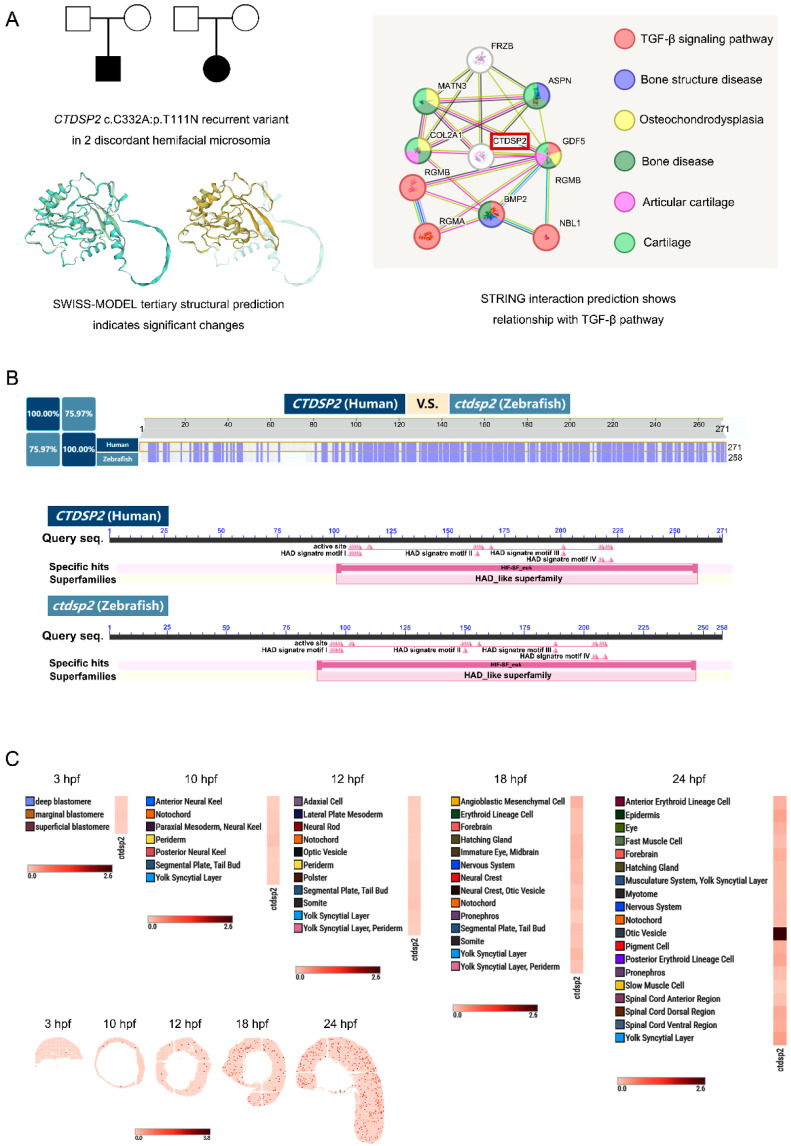
Identification of *CTDSP2* and its homologous gene *ctdsp2* in zebrafish: (**A**) Schematic representation of *CTDSP2* sequence variants in families with hemifacial microsomia. The variant c.C332A:p.T111N was identified in probands with sporadic hemifacial microsomia (black), but it was absent in their unaffected parents (blank). Protein structure predictions suggest significant changes from the wildtype (bottom-left image, left: wildtype CTDSP2; right: mutant type), and pathway analysis indicates an interaction between CTDSP2 and the TGF-β pathway. (**B**) BLAST analysis reveals sequence homology and conserved domain characteristics of zebrafish *ctdsp2*, which is homologous to human *CTDSP2*. (**C**) The expression profile of zebrafish *ctdsp2*, as determined by the Spatial Transcript Omics DataBase, shows dynamic expression patterns during embryonic development.

In our previous whole-exome sequencing (WES) study encompassing 12 individuals with HFM and their unaffected biological parents, we identified a de novo variant of the *CTDSP2* gene, specifically the c.C332A:p.T111N mutation, in two unrelated probands. In silico analysis using SIFT and Polyphen-2 predicted this variant to be “damaging”, suggesting potential pathogenicity. Sanger sequencing verified that the unaffected parents of the probands did not carry this variant. Structural predictions suggested that the variant induces significant alterations in the protein’s structure. Furthermore, protein–protein interaction analysis with STRING software (version 12.0) identified possible associations between CTDSP2 and proteins pivotal to cartilage development, including COL2A1, MATN3, ASPN, and GDF5, as well as components of the TGF-β pathway, namely, BMP2, RGMA, RGMB, and NBL1 (Figure 1A). The TGF-β signaling pathway is crucial for NCC fate regulation, osteoblast differentiation, and bone formation [18,19]. Collectively, these findings underscore the significance of *CTDSP2* in the etiology of HFM.

### 2.2. Homology and Expression Profile of CTDSP2 in Zebrafish

The human CTDSP2 protein, comprising 271 amino acids, features a conserved HAD_SCP1-like domain that includes 18 HAD signature motifs and 18 active sites. A BLAST analysis conducted on the UniProt database (https://www.uniprot.org/ (accessed on 8 July 2022)) identified a zebrafish homolog of CTDSP2, which encodes a 258-amino-acid protein sharing the same conserved domain. The human and zebrafish protein sequences showed a 75.97% sequence identity, indicative of a high degree of homology (Figure 1B).

The spatiotemporal expression pattern of *ctdsp2* during zebrafish embryonic development was investigated using the Zebrafish Embryogenesis Spatiotemporal Transcriptomic Atlas (ZESTA) database (https://db.cngb.org/stomics/zesta/ (accessed on 17 May 2022)). At 10 h post-fertilization (hpf), ctdsp2 expression was detected in the anterior neural keel and notochord. By 18 hpf, the expression was primarily observed in the neural crest and otic vesicle, with continued expression in the otic vesicle by 24 hpf (Figure 1C).

**Figure 2:**
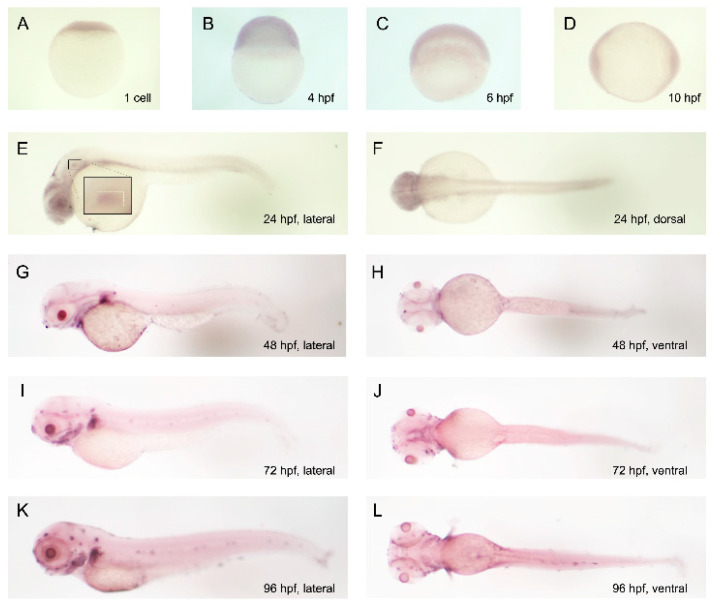
Temporal and spatial expression patterns of *ctdsp2* in developing zebrafish embryos: (**A**−**D**) *ctdsp2* is broadly expressed throughout the entire embryo from the 1-cell stage to 10 h post-fertilization (hpf). (**E**,**F**) By 1 day post-fertilization (dpf), *ctdsp2* expression is notably elevated in the head, eyes, and otic vesicle (inset). (**G**–**L**) From 2 dpf, *ctdsp2* expression becomes concentrated in the mandibular region.

In vivo expression profiling using ISH revealed ubiquitous *ctdsp2* mRNA expression from the 1-cell stage to 10 hpf. By 24 hpf, the expression became more pronounced in the head, eyes, and otic vesicle. By 48 hpf, the expression was notably concentrated in the mandibular region, with intensification observed between 48 and 96 hpf (Figure 2). These findings suggest a specific role for *ctdsp2* in mandibular development, which is in line with its association with pharyngeal arch development.

### 2.3. ctdsp2 Knockout Induces Severe Pharyngeal Malformation

**Figure 3:**
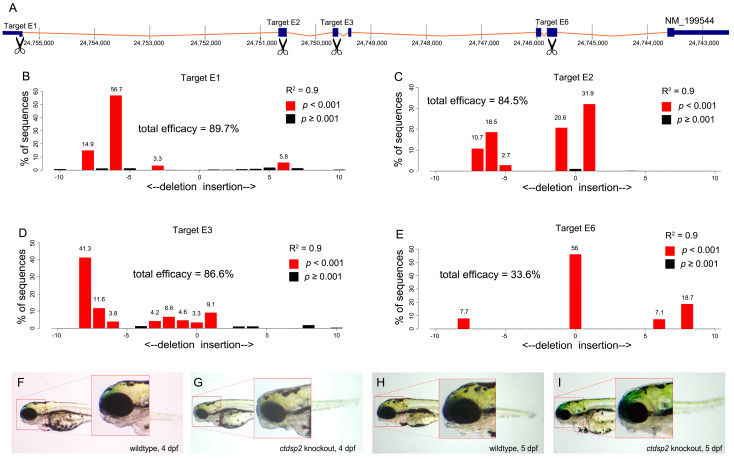
Knockout of *ctdsp2* and associated phenotypic changes: (**A**) Schematic diagram illustrating the four *ctdsp2* gRNA targets in the CRISPR/Cas9 knockdown system. (**B**–**E**) Representative efficiencies of the four gRNA targets in *ctdsp2* knockout. (**F**–**I**) Bright-field images of wildtype and ctdsp2-knockout zebrafish at 4 dpf (**F**,**G**) and 5 dpf (**H**,**I**), demonstrating mandibular deformity in ctdsp2-knockout embryos.

To elucidate the role of *ctdsp2* in craniofacial development, we utilized the CRISPR/Cas9 system to generate a *ctdsp2* knockout in zebrafish. We designed four guide RNAs (gRNAs) targeting exons 1, 2, 3, and 6 of the *ctdsp2* gene (Figure 3A, Supplementary Table S1) and co-injected them with Cas9 protein into one-cell embryos. Sanger sequencing and TIDE analysis confirmed the editing efficiencies of the gRNAs to be 89.7%, 84.5%, 86.6%, and 33.6%, respectively (Figure 3B–E). A substantial proportion of the injected embryos exhibited reduced size of craniofacial structures, mandibular malformations, and microphthalmia at 4 and 5 days post-fertilization (dpf) when compared to the control group (Figure 3F–I).

### 2.4. Inbred Homozygous Zebrafish Demonstrate Similar Pharyngeal Malformations

**Figure 4:**
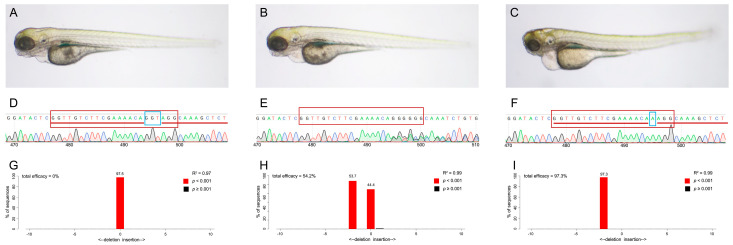
(**A**–**C**) Phenotypes, (**D**–**F**) Sanger sequencing chromatograms, and (**G**–**I**) genotypes of F2 embryos. The F2 generation displayed two distinct phenotypic categories and three different genotypes: embryos without deformities had either wildtype or heterozygous ((−3+1) bp indels) genotypes, while those with deformities were exclusively homozygous for the (−3+1) bp indels. The red box denotes the sequence of gRNA (exon 1) (GGT TGT CTT CGA AAA CAG GT AGG), and the blue box indicates the 3 bp deletion sequence (GGT) and the 1 bp insert (A).

We selected the gRNA with the highest efficacy (E1) to establish a *ctdsp2*-knockout model and evaluate germline transmission. The F0 generation of zebrafish was crossed with Tuebingen (TU) zebrafish. The resulting F1 embryos did not exhibit any distinguishable phenotypes. Upon maturation, F1 mutants were mated to produce F2 offspring, which underwent fin clipping for subsequent sequencing. The F2 embryos presented with two distinct phenotypes: Phenotype 1, resembling wildtype individuals, and Phenotype 2, mirroring the characteristics of the gRNA-injected embryos, in a 3:1 ratio (Figure 4A–C). Genotype analysis indicated that Phenotype 1 embryos were either wildtype or heterozygous, whereas Phenotype 2 embryos were homozygous for the (−3+1) bp indel mutation (Figure 4D–I). The genotype distribution in the F2 embryos, approximating a 1:2:1 ratio, suggested a recessive inheritance pattern of the phenotype.

The impact of the (−3+1) bp indel variant on the secondary and tertiary structures of Ctdsp2 was further assessed, revealing a frameshift mutation that resulted in premature termination and subsequent loss of function (Figure S1A,B). The impact of the (−3+1) bp indel variant on the secondary and tertiary structures of Ctdsp2 was further assessed, revealing a frameshift mutation that resulted in premature termination and subsequent loss of function (Figure S1A,B). The wildtype Ctdsp2 protein, comprising 258 amino acids, was contrasted with the mutant form, which contained only 59 amino acids, significantly altering its tertiary structure (Figure S1C,D).

### 2.5. ctdsp2^−/−^ Embryos Exhibit Craniofacial Malformations, Pharyngeal Arch Cartilage Dysplasia, and Chondrocyte Disorganization

**Figure 5:**
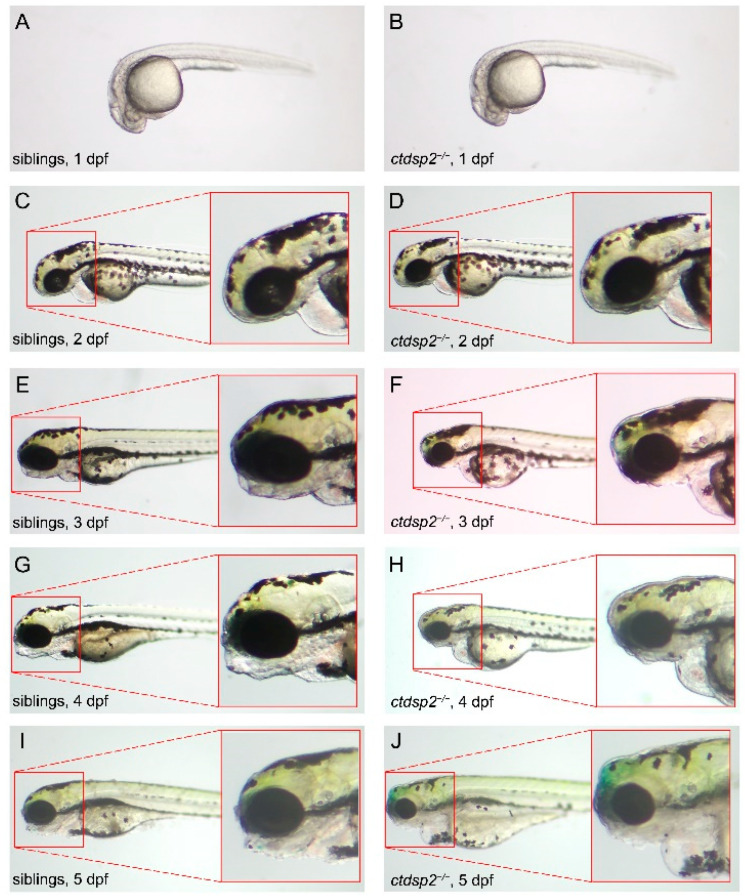
Phenotypic comparison between *ctdsp2*^−/−^ mutants and wildtype zebrafish embryos from 1 to 5 days post-fertilization (dpf): (**A**–**D**) No significant phenotypic differences were observed between the two groups at 1 dpf and 2 dpf. (**E**–**J**) *ctdsp2*^−/−^ embryos abnormally lacked visible signs of mandible development and exhibited cranial developmental abnormalities and microphthalmia compared to wildtypes from 3 dpf. The differences persisted until 5 dpf (**I**,**J**), with all *ctdsp2*^−/−^ embryos dying of swallowing difficulties and cardiac edema.

Initial comparisons between *ctdsp2*^−/−^ embryos and their siblings, including both wildtypes and heterozygotes, during the early developmental stages revealed no significant differences (Figure 5A–D). However, at 3 dpf, *ctdsp2*^−/−^ embryos demonstrated a lack of visible mandible development, accompanied by cranial abnormalities and microphthalmia (Figure 5E,F). These anomalies in mandible structure persisted by 4 dpf, and all *ctdsp2*^−/−^ embryos succumbed to swallowing difficulties and cardiac edema by 5 dpf (Figure 5I,J). Notably, there were no significant differences in embryo size, suggesting that the mutant phenotypes were not a result of developmental delay.

**Figure 6:**
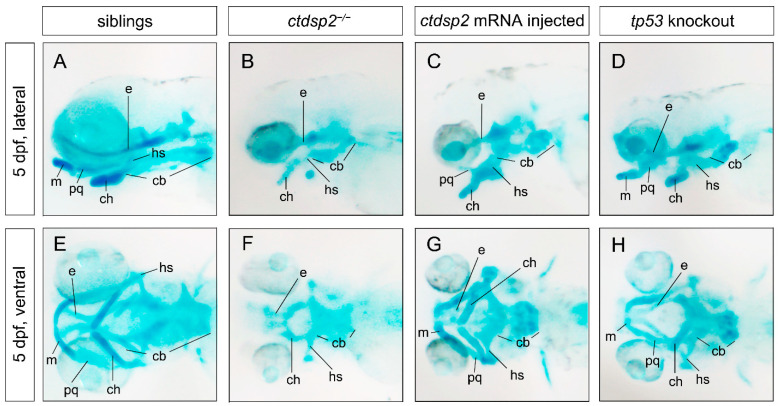
Staining results of pharyngeal arch cartilages using Alcian blue in various groups: (**A**,**E**) Wildtype embryos, (**B**,**F**) *ctdsp2*^−/−^ embryos, (**C**,**G**) *ctdsp2*^−/−^ embryos injected with *ctdsp2* mRNA, and (**D**,**H**) *tp53*-knockout *ctdsp2*^−/−^ embryos. Abbreviations: m, Meckel’s cartilage; pq, palatoquadrate cartilage; ch, ceratohyal cartilage; cb, ceratobranchial cartilage; hs, hyosymplectic cartilage; e, ethmoid plate cartilage.

**Figure 7:**
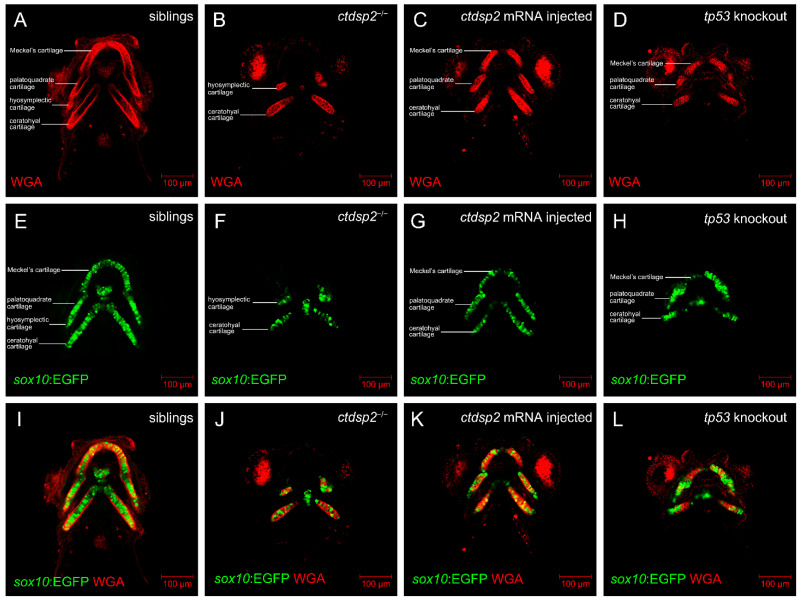
Comparative analysis of chondrocyte morphology in (**A**,**E**,**I**) control siblings, (**B**,**F**,**J**) *ctdsp2*^−/−^ mutants, (**C**,**G**,**K**) *ctdsp2*^−/−^ embryos injected with *ctdsp2* mRNA, and (**D**,**H**,**L**) *tp53*-knockout *ctdsp2*^−/−^ embryos. The craniofacial cartilage in control siblings demonstrates a consistent, elongated, and slender chondrocyte structure, forming a “stack of pennies” organization. In *ctdsp2*^−/−^ embryos, chondrocytes within the cartilage are noticeably smaller, and the overall cartilage structures are markedly deformed. Injection of *ctdsp2* mRNA or knockout of *tp53* in mutants partially rescued chondrocyte morphology.

Alcian staining at 5 dpf in *ctdsp2*^−/−^ embryos revealed an absence of specific cartilaginous structures, including Meckel’s (M) and palatoquadrate (pq), as well as hypoplastic ethmoid plate (e), ceratohyal (ch), hyosymplectic (hs), and ceratobranchial cartilages, when compared to their siblings (Figure 6A,B,E,F). Wheat germ agglutinin (WGA) staining, which visualizes chondrocyte structures, showed a consistent, thin, and elongated structure in siblings at 5 dpf (Figure 7A,E,I). In contrast, *ctdsp2*^−/−^ embryos displayed distorted and disoriented chondrocytes in the deformed hyosymplectic and ceratohyal cartilages (Figure 7B,F,J), indicating severe defects in pharyngeal arch cartilage development. The injection of in vitro synthesized ctdsp2 mRNA into *ctdsp2*^−/−^ embryos rescued the defective pharyngeal arch cartilage phenotypes (Figure 6C,G) and restored the integrity of chondrocyte structure (Figure 7C,G,K).

### 2.6. ctdsp2 Knockout Influences Apoptosis and Proliferation of NCCs in the Pharyngeal Arches and Chondrocyte Differentiation

**Figure 8:**
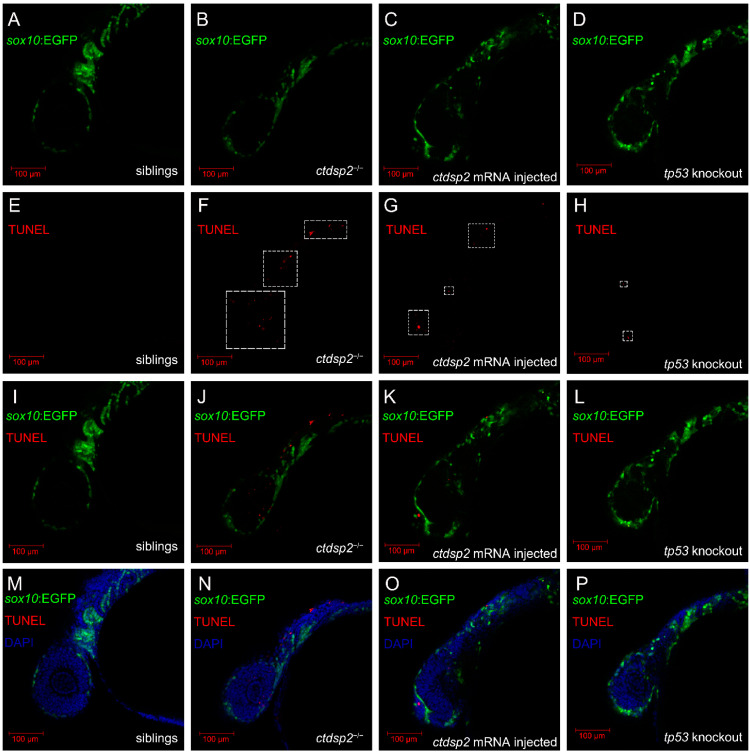
Analysis of somatic cell apoptosis in (**A**,**E**,**I**,**M**) control siblings, (**B**,**F**,**J**,**N**) *ctdsp2*^−/−^ mutants, (**C**,**G**,**K**,**O**) *ctdsp2*^−/−^ embryos injected with *ctdsp2* mRNA, and (**D**,**H**,**L**,**P**) *tp53*-knockout *ctdsp2*^−/−^ embryos at 24 h post-fertilization (hpf). TUNEL staining signals, indicative of apoptotic cells, were observed in the head, pharyngeal arch, and trunk in *ctdsp2*^−/−^ mutants at 24 hpf. The signals were reduced after *ctdsp2* mRNA injection. Control siblings and *ctdsp2*^−/−^ embryos with *tp53* knockout showed no apparent apoptotic signals. The dotted boxes highlight areas of TUNEL staining, emphasizing specific areas where apoptosis was detected.

**Figure 9:**
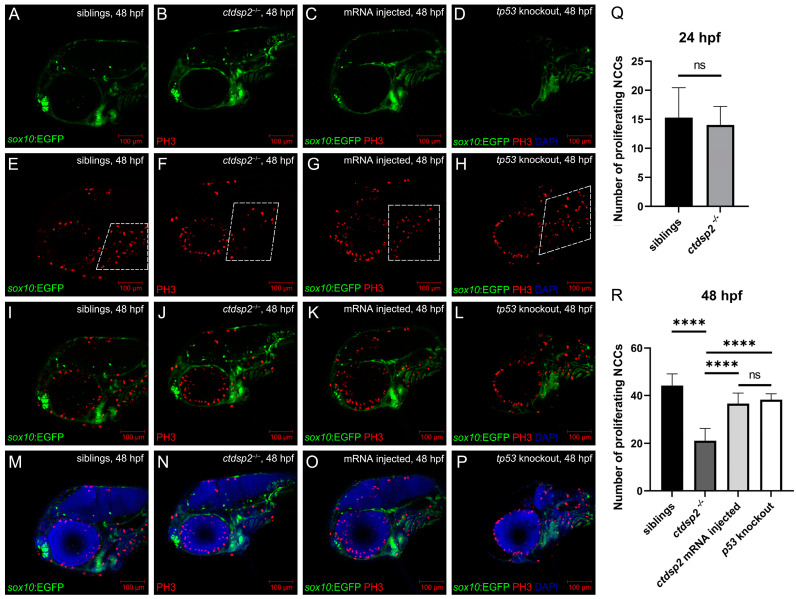
Immunofluorescence results depicting NCC proliferation through antiphosphohistone H3 (PH3) staining in (**A**,**E**,**I**,**M**) control siblings, (**B**,**F**,**J**,**N**) *ctdsp2*^−/−^ mutants, (**C**,**G**,**K**,**O**) *ctdsp2*^−/−^ embryos injected with *ctdsp2* mRNA, and (**D**,**H**,**L**,**P**) *tp53*-knockout *ctdsp2*^−/−^ embryos at 48 h post-fertilization (hpf). The merged images highlight the anti-PH3 signals in the NCCs located in the pharyngeal arch region, marked by a dotted box. (**Q**,**R**) Number of proliferating NCCs among each group. Note: Immunofluorescence experiments were performed in triplicate, statistical analyses were carried out using 3 randomly picked embryos in each group, and statistical significance was assessed by unpaired *t*-tests, with *p* < 0.05 considered statistically significant; **** p < 0.0001.

In light of the observed cartilage malformations, we assessed chondrocyte development from NCCs in *ctdsp2*^−/−^ embryos and their siblings. TUNEL staining detected apoptosis signals among NCCs in *ctdsp2*^−/−^ embryos at 24 hpf (Figure 8B,F,J,N), whereas control siblings exhibited no significant apoptosis (Figure 8A,E,I,M). PH3 staining revealed comparable NCC proliferation in *ctdsp2*^−/−^ embryos and siblings at 24 hpf (15.30 ± 4.86 vs. 14.00 ± 3.03 cells per fish, *p* = 0.5046) (Figure S2). However, a pronounced decrease in NCC proliferation was observed in *ctdsp2*^−/−^ mutants compared to siblings at 48 hpf (44.20 ± 4.62 vs. 21.10 ± 4.97 cells per fish, **** p < 0.0001) (Figure 9).

**Figure 10:**
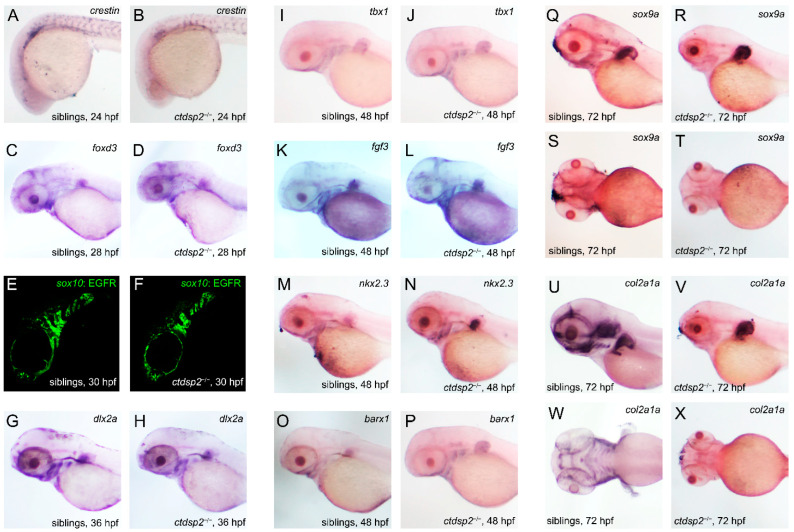
The impact of *ctdsp2* knockout on pharyngeal pouches, neural crest cells (NCCs), and pharyngeal cartilage development in zebrafish embryos: (**A**–**D**) ISH results with crestin and *foxd3* probes, markers for NCCs, at 24 h post-fertilization (hpf) and 28 hpf. The staining patterns show no noticeable differences between mutant embryos and their control siblings. (**E**,**F**) Fluorescence imaging of *sox10*-labeled NCCs in the pharyngeal arch region. Green fluorescence signals indicating NCCs are similar in both *ctdsp2*^−/−^ embryos and siblings. (**G**,**H**) Expression of *dlx2a* at 30 hpf appears similar in both *ctdsp2*^−/−^ and control embryos. (**I**–**N**) Expression of *tbx1*, *fgf3*, and *nkx2.3* at 48 hpf shows no significant differences in the segmentation and number of pharyngeal pouches between *ctdsp2*^−/−^ embryos and siblings. (**O**,**P**) ISH with the *barx1* probe at 48 hpf. No significant variation was observed between mutant and control embryos. (**Q**–**X**) ISH with *sox9a* and *col2a1a* probes at 72 hpf; *sox9a* expression is notably reduced in mutants, and the expression of *col2a1a*, a marker for cartilage, is absent in the hypopharyngeal arches of the mutant embryos compared to controls.

**Figure 11:**
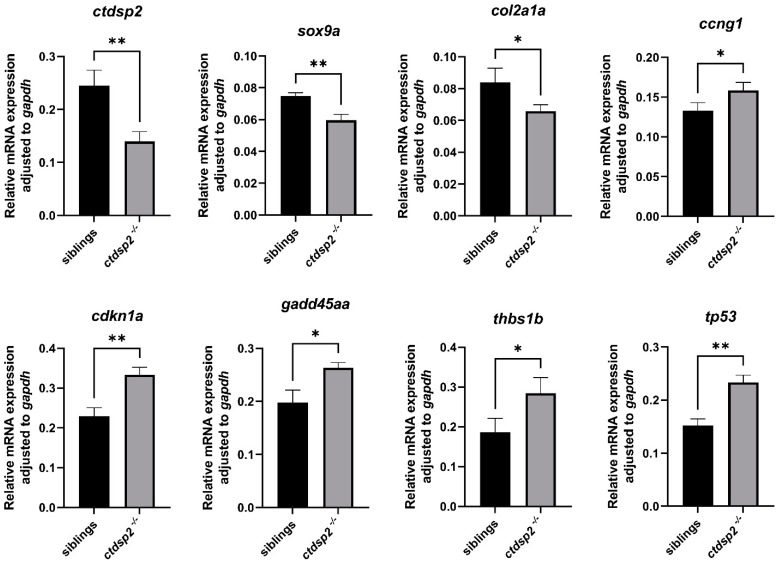
qPCR analysis of gene expression in *ctdsp2*^−/−^ mutant and control sibling zebrafish embryos. Note: The comparison was conducted using an unpaired t-test, and a *p*-value less than 0.05 was considered a significant difference; * *p* < 0.05, ** *p* < 0.01.

To evaluate the effects of *ctdsp2* knockout on NCC development stages, we performed ISH assays on marker genes at various developmental stages. The NCC migration and specification markers, crestin and *foxd3*, showed no significant differences at 24 hpf and 28 hpf, suggesting no impact on NCC formation (Figure 10A–D). Transgenic fish Tg(*sox10*:EGFP) labeled NCCs, exhibiting similar green fluorescence signals in the pharyngeal arch region of both mutants and siblings at 30 hpf (Figure 10E,F), indicating unaffected NCC migration. The *dlx2a* expression domains at 30 hpf were comparable, suggesting no specialization impact (Figure 10G,H). The marker genes *tbx1*, *fgf3*, and *nkx2.3* showed no significant differences in segmentation or number of pharyngulae, suggesting no influence on pharyngula formation (Figure 10I–N). No significant differences in *barx1* expression were observed between mutants and siblings at 48 hpf (Figure 10O,P), indicating that *ctdsp2* knockout did not affect mesenchymal cell aggregation in the pharyngeal arch primordium. However, the expression of *sox9a* and *col2a1a*, which are co-expressed during chondrogenesis, was significantly downregulated in *ctdsp2*^−/−^ mutants at 72 hpf, suggesting a role for Ctdsp2 in zebrafish chondrogenic differentiation. Additionally, qRT-PCR analysis revealed lower mRNA levels of *ctdsp2*, *sox9a*, and *col2a1a* in *ctdsp2*^−/−^ embryos compared to controls (Figure 11, ** *p* = 0.0063, ** *p* = 0.0034, and * *p* = 0.0342).

### 2.7. RNA-Seq Analysis Identifies Differentially Expressed Genes and Associated Signaling Pathways

To elucidate the molecular phenotypes of *ctdsp2*^−/−^ embryos, a comprehensive transcriptome analysis was conducted. Principal component analysis (PCA) distinguished clear clusters between the control and mutant groups, signifying substantial molecular differences. A total of 2345 differentially expressed genes (DEGs) were identified, with 1224 upregulated and 1121 downregulated in the mutants, all exhibiting fold changes greater than 1.0 and *p*-values less than 0.05 (Figure S3 and Table S2). KEGG enrichment analysis of these DEGs indicated a significant enrichment of genes involved in the p53 signaling pathway, which is intimately linked with zebrafish maxillofacial development (Figure S4). Several DEGs within the p53 pathway, including *baxa*, *casp8*, *casp8l2*, *ccng1*, *cdkn1a*, *gadd45aa*, *gadd45ga*, *mdm2*, *serpine1*, *sesn2*, *thbs1b*, and *tp53*, were found to be upregulated in the mutants. qRT-PCR analysis confirmed significantly higher expression of *ccng1*, *cdkn1a*, *gadd45aa*, *thbs1b*, and *tp53* in *ctdsp2*^−/−^ embryos (Figure 11; * *p* = 0.0343, ** *p* = 0.003, * *p* = 0.0106, * *p* = 0.0321, and ** *p* = 0.0016), while the expression levels of the remaining genes showed no significant differences (Figure S5).

### 2.8. tp53 Knockout Alleviates Pharyngeal Arch Cartilage Dysplasia in ctdsp2−/− Embryos

To elucidate the role of the p53 signaling pathway in the observed phenotypes, we conducted knockout studies targeting the *ccng1*, *cdkn1a*, *gadd45aa*, *thbs1b*, and *tp53* genes in *ctdsp2*^−/−^ embryos and evaluated their impact on pharyngeal arch cartilage dysplasia. Alcian blue staining revealed that only the knockout of *tp53* partially restored the defective cartilage phenotypes in *ctdsp2*^−/−^ mutants. The rescued embryos showed redeveloped Meckel’s and palatoquadrate cartilages, along with a thickening of the hyosymplectic cartilage, albeit with an abnormal angle (Figure 6D,H). WGA staining corroborated these findings, demonstrating clear restoration of Meckel’s cartilage and chondrocyte morphology in the rescued embryos (Figure 7D,H,L). TUNEL staining and PH3 immunostaining indicated a significant reduction in cell apoptosis (Figure 8D,H,L,P) and a restoration of the proliferation signal of NCCs in the pharyngeal arch of *tp53*-knockout *ctdsp2*^−/−^ embryos (Figure 9D,H,L,P).

## 3. Discussion

The etiology of HFM is multifaceted, encompassing both environmental and genetic factors. Our initial study identified a potential pathogenic variant (c.C332A:p.T111N) in *CTDSP2*, which has not previously been associated with HFM [14]. The stable zebrafish lines with *ctdsp2* knockout, established using CRISPR/Cas9, exhibited craniofacial malformations that closely resemble HFM in humans. In terms of pathogenic mechanisms, both in silico and in vivo experiments have revealed the association of *CTDSP2* with chondrogenic differentiation of NCCs as well as the TGF-β and p53 signaling pathways. The observed phenotypes and functional studies in *ctdsp2*^−/−^ zebrafish support the hypothesis that *CTDSP2* plays a regulatory role in craniofacial development, underscoring its significance in this process.

The classical hypotheses regarding HFM’s etiology include vascular anomalies, damage to Meckel’s cartilage, and abnormalities in NCC development [17]. Our findings suggest that *ctdsp2* may not be essential for NCCs’ migration, aggregation, or specification but is crucial for their differentiation into chondrocytes in zebrafish, as predicted by bioinformatics analysis and demonstrated by the downregulation of sox9a and col2a1a in *ctdsp2*^−/−^ mutants. *Sox9* is believed to be essential for chondrogenesis by regulating the expression of *Col2a1* [20], the product of which, collagen II, is a prominent component of the cartilage extracellular matrix [21]. Similar phenomena of abnormal chondrogenic differentiation of NCCs have been observed in previous HFM zebrafish models, such as those with knockouts of *cdc27*, *fbln2*, and *amer1*, suggesting a potential commonality in the pathogenesis of HFM, which warrants further investigation with additional candidate genes [14,22,23].

The reduction in NCC proliferation and increase in apoptosis in *ctdsp2*^−/−^ embryos are associated with various craniofacial abnormalities. Increased apoptosis in NCCs has been linked to impaired secondary palate fusion and severe midface abnormalities, including cleft palate [24,25]. Reduced NCC proliferation due to variants in *VWA1*, *CDC27*, *AMER1*, and *FBLN2* has been implicated in HFM, and zebrafish models carrying mutations in these genes presented similar malformations of craniofacial structures [14,22,23,26]. Mechanistically, *CTDSP2* has been shown to be an important regulator of the cell cycle by modulating *P21*^Cip1/Waf1^ via Ras signaling [16], through which its variants could be associated with cell proliferation and apoptosis. Dysregulation of NCC proliferation and apoptosis tends to result in a reduction in chondrogenic progenitors in the pharyngeal arch mesenchyme, potentially contributing to craniofacial developmental abnormalities.

RNA sequencing analysis revealed an enrichment of highly expressed genes related to the p53 signaling pathway in *ctdsp2*^−/−^ embryos. Moreover, the partial rescue of the phenotype in *ctdsp2*^−/−^ embryos upon *tp53* knockout suggests that aberrant activation of the p53 pathway may be associated with the pathogenesis of HFM. Aberrant activation of the p53 pathway has been associated with various congenital disorders involving craniofacial malformations, such as Treacher Collins syndrome (TCS), as demonstrated by a *Tcofl1*+/− mouse model [27]. Additionally, patients with POLR1C and POLR1D mutations exhibit increased NCC apoptosis, which can be rescued by *tp53* knockout in zebrafish [28,29]. Therefore, the p53 pathway appears to be a significant factor in the pathogenesis of craniofacial malformations; p53 activation can induce cell-cycle arrest, inhibiting proliferation and elevating apoptosis [30]. Accordingly, the dysregulation of NCC proliferation and apoptosis in *ctdsp2*^−/−^ embryos was ameliorated by *tp53* knockout, supporting the involvement of the p53 pathway in the regulation of these cellular behaviors.

However, while the p53 pathway is believed to suppress stemness, the NCCs of *ctdsp2*^−/−^ embryos displayed abnormal chondrogenic differentiation that could not be rescued by *tp53* knockout, indicating the complexity of HFM’s pathogenesis induced by *CTDSP2* variants. Our bioinformatics analysis revealed potential interactions between CTDSP2 and proteins in the TGF-β signaling pathway, suggesting the potential involvement of TGF-β, in addition to p53, in the pathogenesis of *CTDSP2* variants. The TGF-β pathway is considered to play a pivotal role in chondrogenic differentiation of NCCs by promoting *Sox9* expression while suppressing *Msx1/2* expression [20,31]. Moreover, previous studies have shown that the proliferation and apoptosis of NCCs are also associated with this pathway [31,32]. Therefore, TGF-β dysregulation might partially contribute to the abnormal development of NCCs in *ctdsp2*^−/−^ embryos and the pathogenesis of HFM. Notably, TGF-β has been implicated in other animal models of craniofacial malformations, such as *fbln2*-knockout zebrafish and *Tgfbr2*-mutant mice [33], indicating that TGF-β signaling may be a common factor in the pathogenesis of these disorders.

While this study provides valuable insights into the etiology and pathogenesis of HFM, it is not without limitations. Firstly, the suspected pathogenic non-synonymous variants of *CTDSP2* were identified in only two unrelated probands, necessitating further research with larger sample sizes to confirm the pathogenicity of this site. Additionally, while the zebrafish model offers valuable insights, the lack of functional validation in mammalian models, particularly those closely resembling human facial development, remains a limitation. Furthermore, the pathogenic mechanism behind *CTDSP2* variants appears to be complex, as indicated by the only partial phenotypic amelioration observed through *tp53* knockout in *ctdsp2*^−/−^ embryos. Other factors, such as the TGF-β pathway, are also potentially involved, yet their association with *CTDSP2* variants and HFM’s pathogenesis still lacks experimental evidence.

## 4. Materials and Methods

### 4.1. Zebrafish and Embryos

All zebrafish experiments were conducted with the approval of the Animal Ethics Committee at Peking University, under protocol code LSC-ZhangB-3, and were granted approval on 1 September 2019. The experiments complied with all relevant institutional, regional, and national regulations and guidelines. The Tuebingen and transgenic fish lines, including the Tg (*sox10*: EGFP) line (ID: CZ156, ba2Tg/+) [34], were sourced from the China Zebrafish Resource Center (CZRC) (http://www.zfish.cn/ (accessed on 18 March 2021)). Adult zebrafish were maintained under a controlled environment at 28.5 °C with a 14 h light and 10 h dark cycle, and embryonic development was staged according to standard methodologies [35]. To inhibit pigment formation, the zebrafish embryos were treated with 0.2 mM 1-phenyl-2-thio-urea (PTU) at 24 hpf.

### 4.2. Zebrafish Target Gene Knockout

We employed the CRISPR/Cas9 system to achieve targeted gene knockout in zebrafish, adhering to a well-established rapid method for directed gene knockout in F0 zebrafish [36]. The design of guide RNAs (gRNAs) was facilitated by the CRISPR Design Website (http://chopchop.cbu.uib.no/), accessed on 9 July 2022. The pMD19-gata5_gRNA scaffold served as the template for gRNA production. The sequences of the gRNAs utilized are detailed in Table S1. Four gRNAs, along with the Cas9 protein, were co-injected into one-cell stage embryos. The efficacy of the gRNAs was validated by extracting crude genomic DNA from both the control group, consisting of TU zebrafish embryos, and the experimental zebrafish embryos.

### 4.3. Bioinformatics Prediction of ctdsp2 Expression Profile in Zebrafish

The Spatial Transcript Omics DataBase (STOmics DB) was utilized to assess the expression profile of the *ctdsp2* gene during the early stages of zebrafish embryonic development. This analysis was conducted using stereo-sequencing (stereoseq) data, accessed on 17 June 2023, through the STOmics DB portal (https://db.cngb.org/stomics/) [37]. Bin annotation techniques were employed to analyze the stereoseq data, facilitating a comprehensive understanding of *ctdsp2* gene expression patterns.

### 4.4. Whole-Mount In Situ Hybridization

We conducted whole-mount ISH on zebrafish embryos in accordance with established protocols [37]. To generate the *ctdsp2* antisense probe, we amplified the full-length zebrafish *ctdsp2* gene using specific primers: a forward primer (5′-GCA CAG CTC CTT TAA GCC AA-3′) and a reverse primer (5′-TAA TAC GAC TCA CTA TAG GGC TCA AGG ATG AAC GGG TGC T-3′) that included the T7 polymerase sequence. The antisense probe was subsequently synthesized utilizing T7 polymerase and a digoxin–NTP mixture (Dig-NTP mix, Roche, Basel, Switzerland). Additionally, probes targeting *crestin*, *foxd3*, *dlx2a*, *tbx1*, *fgf3*, *nkx2.3*, *barx1*, *sox9a*, and *col2a1a* were used in the whole-mount ISH (Table S3). Zebrafish embryos at specific developmental stages were collected and fixed in 4% paraformaldehyde (PFA) to preserve tissue structure and mRNA integrity. The fixed samples were permeabilized with proteinase K to enable probe penetration. A labeled probe was then added, and the hybridization reaction was carried out at around 65 °C for several hours to overnight. After hybridization, the samples were extensively washed with buffers of different stringencies to eliminate unbound probes and reduce background noise. Next, an alkaline phosphatase-conjugated anti-digoxigenin antibody was added to bind to the labeled probe, followed by the introduction of a chromogenic substrate. The enzyme on the antibody reacted with the substrate, producing a colored precipitate at the probe–target mRNA binding sites, thus visualizing the gene expression pattern.

### 4.5. Cartilage Staining

To visualize cartilaginous structures within zebrafish embryos, we employed Alcian blue staining to highlight the cartilage architecture, complemented by WGA staining to label chondrocyte membranes at the 5 dpf stage. The Alcian blue staining procedure commenced with eight hourly washes in 0.1% Tween-20 H_2_O, followed by an overnight incubation in 0.015% Alcian blue solution (Sigma-Aldrich, Shanghai, China) at 4 °C. Post-staining, the embryos underwent a rehydration process through a gradient of alcohol concentrations, concluding with a transition to distilled water. Subsequently, the samples were treated with 0.25% trypsin at ambient temperature to achieve tissue transparency, following the method described in [34]. For the visualization of chondrocyte membranes, the embryos were immersed in a 1:200 dilution of Alexa Fluor 594-conjugated WGA (Invitrogen, Carlsbad, CA, USA) and incubated overnight at 4 °C, followed by thorough washing with PBST.

### 4.6. Immunofluorescence

We utilized terminal deoxynucleotidyl transferase dUTP nick-end labeling (TUNEL) assays to evaluate cell apoptosis, adhering to the manufacturer’s guidelines for the In Situ Cell Death Detection Kit, TMR red (Roche, Shanghai, China). To indicate cell proliferation, anti-PH3 staining was applied. Transgenic Tg(*sox10*:EGFP) zebrafish embryos were fixed in 4% paraformaldehyde and subsequently washed with PBST. The immunostaining procedure was carried out using PH3 antibodies (1/400 dilution; sc-374669, Santa Cruz Biotechnology, Shanghai, China). For PH3 immunofluorescence staining, the embryos were incubated overnight at 4 °C with the diluted anti-PH3 primary antibody. After washing with PBST, a fluorescence-labeled secondary antibody was added and incubated for 2 to 3 h at room temperature in the dark. DAPI was used for nuclear staining during the final wash. For the TUNEL assay, the TUNEL reaction mixture from a commercial kit was prepared. The zebrafish embryos were incubated with this mixture at 37 °C for 1 to 2 h in the dark. For both apoptosis and proliferation analyses, only cells that were doubly positive for anti-PH3/TUNEL and EGFP were manually enumerated to verify their status as neural crest cells (NCCs). All immunofluorescence images were captured using a Carl Zeiss LSM710 Confocal Microscope (Carl Zeiss AG, Jena, Germany), ensuring uniform settings across all experiments.

### 4.7. RNA Sequencing

Zebrafish embryos at 3 dpf, exhibiting mutant and normal phenotypes, were euthanized for total RNA extraction using TRIzol Reagent (Invitrogen, Waltham, MA, USA). Each experimental group comprised three independent samples, with each sample containing ten embryos. The procedures for assessing sample quality, RNA library preparation, and RNA sequencing (RNA-Seq) were carried out as detailed in a previous study [38]. Consequently, six sequencing libraries were constructed and subjected to sequencing analysis.

The bioinformatics analysis of RNA-Seq data was performed using standard methodologies. Clean reads were aligned to the reference genome, Danio rerio GRCz11, from the NCBI assembly database using HISAT2 (version 2.1.0), resulting in BAM files for subsequent alignment [39]. The DESeq2 package from Bioconductor was utilized for differential gene expression analysis [40]. Genes exhibiting a fold change of ≥1.5 and a *p*-value < 0.05 were classified as differentially expressed genes (DEGs). DEG enrichment analysis was conducted using the KOBAS-i tool, focusing on the Kyoto Encyclopedia of Genes and Genomes (KEGG) and Gene Ontology (GO) databases [41]. KEGG and GO enrichment results, with a *p*-value threshold of <0.05, were generated using the ggplot2 package in RStudio. Jaccard coefficients were calculated in RStudio (Version 1.4.1717) for comparative analysis. To analyze key signaling pathways and genes, the cytoHubba plugin in Cytoscape (Version 3.9.1) was employed, applying the maximal clique centrality (MCC) method [42].

### 4.8. Quantitative Real-Time PCR Validation

To substantiate the findings from the RNA sequencing analysis, quantitative real-time PCR (qPCR) was conducted using well-established methodologies. The primers utilized, along with sequence IDs, gene names, and amplicon lengths, are detailed in the Supplementary Materials (Table S4). The analysis of qRT-PCR data adhered to the protocol outlined by Hellemans et al. [43].

### 4.9. Statistical Analysis

All experimental procedures were carried out in triplicate to ensure reproducibility, and data analysis was conducted using unpaired *t*-tests. Statistical significance was set at the *p* < 0.05 threshold. Consistent results were observed in more than 85% of the embryos across all phenotypic observation experiments, with the selected images being representative of these consistent findings.

## Data Availability

The data presented in this study are available in the article and the Supplementary Materials.

## Supplementary Materials

The following supporting information can be downloaded at https://www.mdpi.com/article/10.3390/ijms26031297/s1.

## Abbreviations

China Zebrafish Resource Center, CZRC; differentially expressed genes, DEGs; Gene Ontology, GO; guide RNA, gRNA; hemifacial microsomia, HFM; hours post-fertilization, hpf; insertions or deletions, indels; in situ hybridization, ISH; Kyoto Encyclopedia of Genes and Genomes, KEGG; maximal clique centrality, MCC; neural crest cell, NCC; 1-phenyl-2-thio-urea, PTU; phosphohistone H3, PH3; principal component analysis, PCA; quantitative real-time PCR, qPCR; terminal deoxynucleotidyl transferase dUTP nick-end labeling, TUNEL; Treacher Collins syndrome, TCS; Tuebingen, TU; wheat germ agglutinin, WGA.
