# Supplementary material for: ctdsp2 Knockout Induces Zebrafish Craniofacial Dysplasia via p53 Signaling Activation
Source: Int J Mol Sci. 2025 Feb 3;26(3):1297. doi: 10.3390/ijms26031297 (PMC11818092; doi:10.3390/ijms26031297)
Supplement: Supplementary file 1 [file ijms-26-01297-s001.zip › Table S1-The gRNAs and related primer sequences.pdf]

Table S1. The gRNAs and related primer sequences

Primers	Sequence（5'→3'）
T7-*ctdsp2* (Exon-1)-sfd	TAATACGACTCACTATAGGTTGTCTTCGAAAACAG GT GTTTTAGAGCTAGAAATAGC
T7- *ctdsp2* (Exon-2)-sfd	TAATACGACTCACTATAgTGCTAGAGCCTGAGGAC AA GTTTTAGAGCTAGAAATAGC
T7- *ctdsp2* (Exon-3)-sfd	TAATACGACTCACTATAGCTGGCAGCAGACTCTC GCC GTTTTAGAGCTAGAAATAGC
T7- *ctdsp2* (Exon-7)-sfd	TAATACGACTCACTATAGTCGTGGTTTGACGATTC GG GTTTTAGAGCTAGAAATAGC
T7- *tp53* (Exon-2)-sfd	TAATACGACTCACTATAGGCTCGAACCACGGAGC CCTGGTTTTAGAGCTAGAAATAGC
tracr rev	AAAAAAAGCACCGACTCGGTGCCAC
ctdsp2 (Exon-1)-Forward	TTGGTGGTGTCTTTTCCCGA
ctdsp2 (Exon-1)-Reverse	CAATGCTTGACTGCAACGGAA
ctdsp2 (Exon-2)-Forward	GTGCCATCTCTGGAAAGGGG
ctdsp2 (Exon-2)-Reverse	GTCAACCAATCCTTGCCACTG
ctdsp2 (Exon-3)-Forward	GATCACTTGCTTACGTCATTCCC
ctdsp2 (Exon-3)-Reverse	GCACTGCTGAAGTGTCGTTC
ctdsp2 (Exon-7)-Forward	ATTTCAGACCTTGCACTCCCA
ctdsp2 (Exon-7)-Reverse	GTTGTGCGGAGCTGAGTAAC
tp53 (Exon-2)- Forward	GGCCATTGTCATTTTTCGTATT
tp53 (Exon-2)- Reverse	AATTACACACACTTGCCTCCCT

Note: The black underline indicates the T7 promoter sequence; The blue sequence indicates the main sequence of the target (excluding PAM); The red sequence indicates the fixed sequence of gRNA scaffold; The "g" refers to the adjustment for the first base of the main sequence is not "g".
