# Supplementary material for: ctdsp2 Knockout Induces Zebrafish Craniofacial Dysplasia via p53 Signaling Activation
Source: Int J Mol Sci. 2025 Feb 3;26(3):1297. doi: 10.3390/ijms26031297 (PMC11818092; doi:10.3390/ijms26031297)
Supplement: Supplementary file 1 [file ijms-26-01297-s001.zip › Table S3-Probe primers of in situ hybridization.pdf]

Table S3. Probe primers of *in situ* hybridization

**Primers**	**Sequence（5'→3'）**
ctdsp2-F	GCACAGCTCCTTTAAGCCAA
ctdsp2-R	CTCAAGGATGAACGGGTGCT
ctdsp2-T7 R	TAATACGACTCACTATAGGGCTCAAGGATGAACGGGTGCT
crestin-F	GAGAAGCCCTCATCAGAGAGTTTG
crestin-R	GTTGCTTGTCAGGCAGAATCAGG
crestin-T7 R	TAATACGACTCACTATAGGGGTTGCTTGTCAGGCAGAATCAGG
foxd3-F	CCTACTCGTACATCGCCCTC
foxd3-R	CCGGGTTAAGGACAGGGAC
foxd3-T7 R	TAATACGACTCACTATAGGGCCGGGTTAAGGACAGGGAC
dlx2a-F	CACAGTTCTGCTTTGCGTCG
dlx2a-R	CCCAAGTCGGCAGAGTCAAA
dlx2a-T7 R	TAATACGACTCACTATAGGGCCCAAGTCGGCAGAGTCAAA
tbx1-F	GCAGCTGTCCCATTTTTGCG
tbx1-R	ACGGCGGTAAATCTGGTCTC
tbx1-T7-R	TAATACGACTCACTATAGGGACGGCGGTAAATCTGGTCTC
fgf3-F	GAATCTCTGGCTCCGAGGCT
fgf3-R	GCCGCTGACTCTCTCTAAGC
fgf3-T7-R	TAATACGACTCACTATAGGGGCCGCTGACTCTCTCTAAGC
nkx2.3-F	TCGTGTTTTCTCGGAGGTGG
nkx2.3-R	GCGCATTAGTGGACGTGTTC
nkx2.3-T7-R	TAATACGACTCACTATAGGGGCGCATTAGTGGACGTGTTC
barx1-F	CTGGGCGGATCAGACTTCTC
barx1-R	GCTTCTCGTGTCCTCTCCTG
barx1-T7 R	TAATACGACTCACTATAGGGGCTTCTCGTGTCCTCTCCTG
sox9a-F	CCTCGACCCCTACCTGAAGA
sox9a-R	GGCGGGAGGTATTGGTCAAA
sox9a-T7 R	TAATACGACTCACTATAGGGGGCGGGAGGTATTGGTCAAA
col2a1a-F	TCTGAAGTCCATCAACGGGC
col2a1a-R	TTTTCCGTCACGCTAAACGC
col2a1a-T7 R	TAATACGACTCACTATAGGGTTTTCCGTCACGCTAAACGC
