# Supplementary material for: ctdsp2 Knockout Induces Zebrafish Craniofacial Dysplasia via p53 Signaling Activation
Source: Int J Mol Sci. 2025 Feb 3;26(3):1297. doi: 10.3390/ijms26031297 (PMC11818092; doi:10.3390/ijms26031297)
Supplement: Supplementary file 1 [file ijms-26-01297-s001.zip › Table S4-Primers of qRT-PCR.pdf]

Table S4. Primers of qRT-PCR

Primers	Sequence（5’→3’）
gapdh-F	GGGTGATGCAGGTGCTACTT
gapdh-R	AAAGGAGCCAGGCAGTTGG
ctdsp2-F	AGGGCTGTGCGTTTGTTAGA
ctdsp2-R	GCAAGGCATCATGGGTAGGT
sox9a-F	TTTGACCAATACCTCCCGCC
sox9a-R	AGGAACCGTAGCTGATGTGC
col2a1a-F	TGACCAGGAAGAGTTTGGCG
col2a1a -R	TTTGCACCAAGTGACCCGAT
ccng1-F	CGATACACTGCAAGCTGAAACTG
ccng1-R	GCTGGAAGAGGTCGTGATTGA
cdkn1a-F	GAGCTTCGACTTTGCGTCTG
cdkn1a-R	CTTGGCTTGGTAGAAATCTGTG
gadd45aa-F	GACCCTGATAACGTGGTGCT
gadd45aa-R	GCATGTGGAACCGTGACCA
thbs1b-F	GGGATGTGAGGAGGTGAACG
thbs1b-R	TCCAGCGTGGTTCCGAATAC
tp53-F	GTGGCTCTTGCTGGGACATC
tp53-R	CAGGTCCGGTGAATAAGTGCAA
baxa-F	TACTTTGCCTGTCGCCTTGT
baxa-R	AGCGAGGAAAACTCCGACTG
casp8-F	TGGGCCAGACAATCTGGATG
casp8-R	CTCCTCCTTTCTCTCTCCATTGT
casp8l2-F	GTCCATCTGCGATCAGGACC
casp8l2-R	CCTCTGCAGGACGTCCAAAT
gadd45ga-F	GCGAAACGCGACCTAAAGTG
gadd45ga-R	ACGCTGTCTGGGTCAACATT
mdm2-F	CCCAGAGCTCAAGCACTTTATGT
mdm2-R	CCTGCATCCTCTAACAGGCTT
serpine1-F	GGTGCATTGCTCTTTAGCGG
serpine1-R	GGCAGTCCAAGCTATGCTGA
sesn2-F	ACGAAGCAGATGACCTCCCT
sesn2-R	GGATGAAGCCCCATCACCAT
