# Supplementary figures and images for: ctdsp2 Knockout Induces Zebrafish Craniofacial Dysplasia via p53 Signaling Activation
Source: Int J Mol Sci. 2025 Feb 3;26(3):1297. doi: 10.3390/ijms26031297 (PMC11818092; doi:10.3390/ijms26031297)

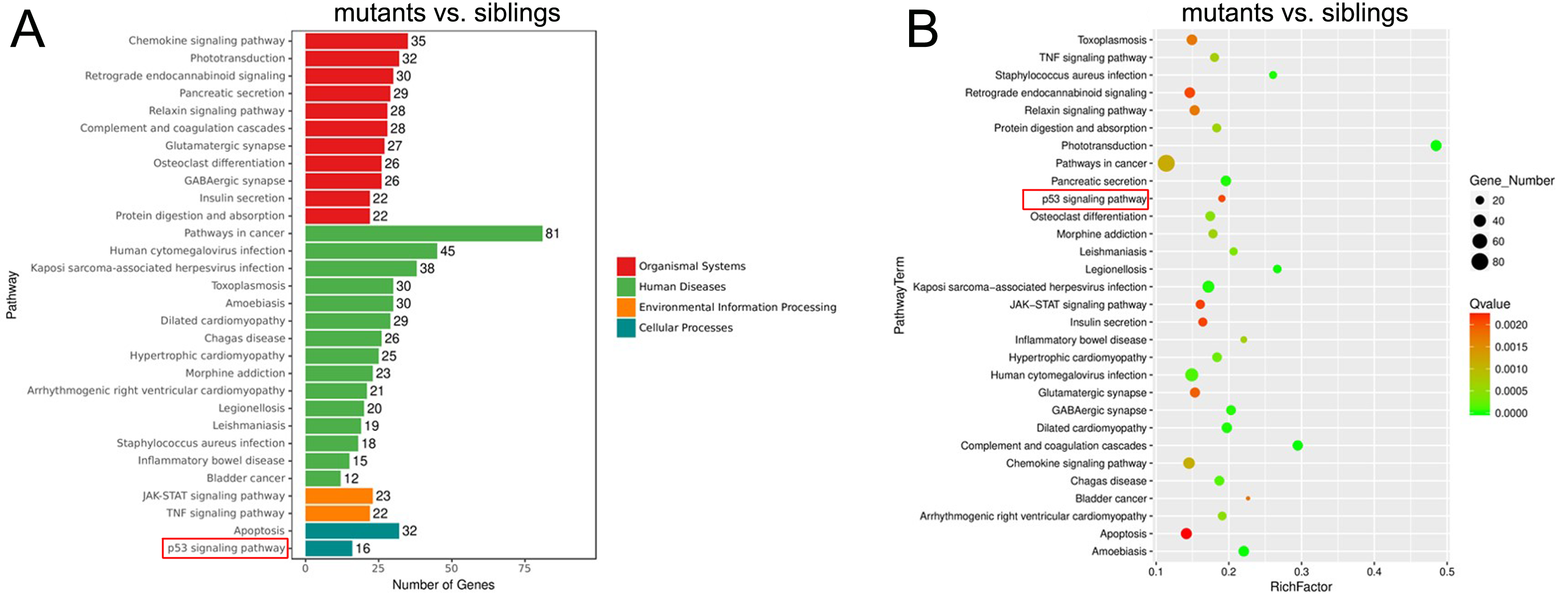

Supplement: Supplementary file 1 [file ijms-26-01297-s001.zip › figure S4.tif]

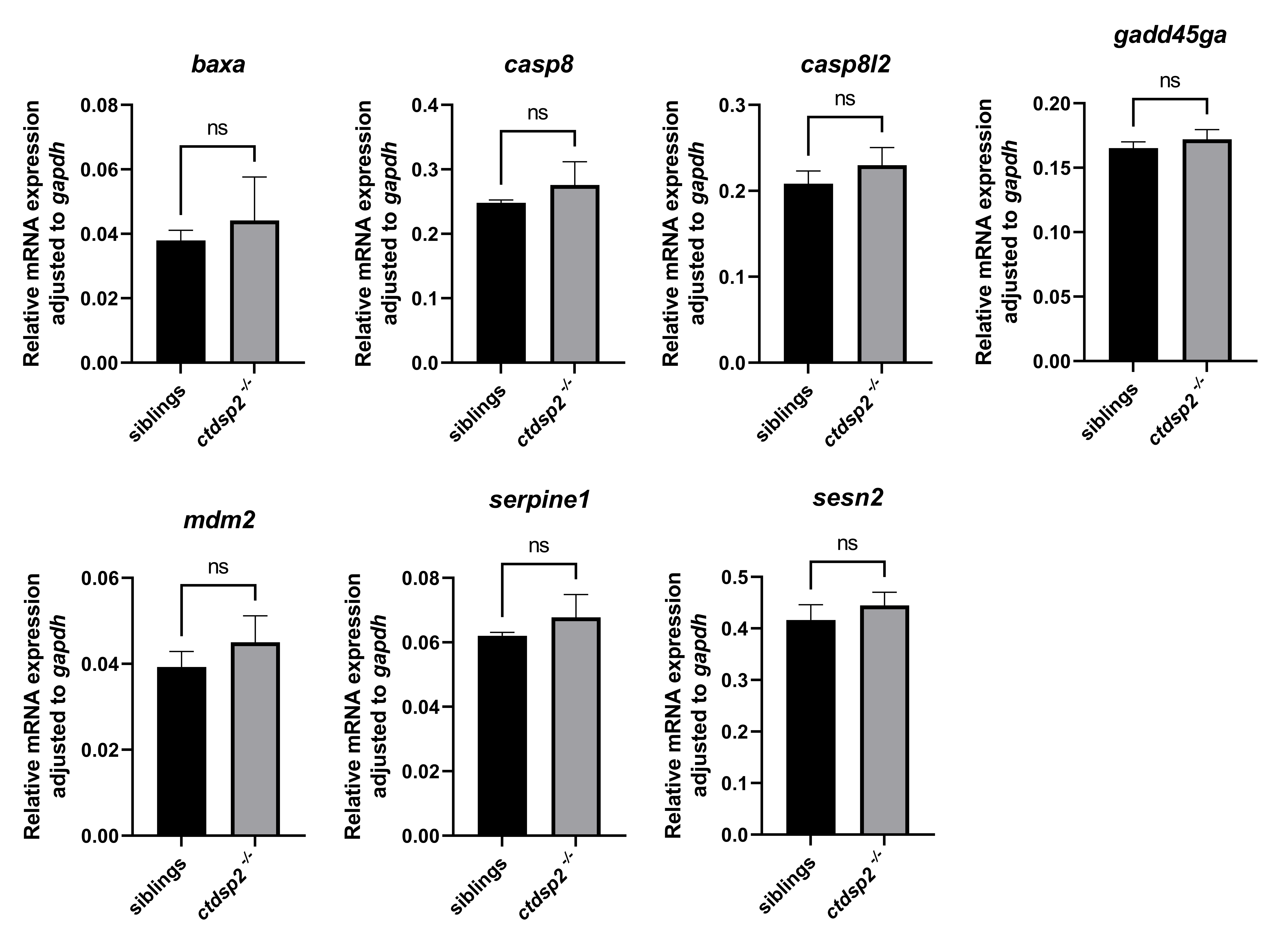

Supplement: Supplementary file 1 [file ijms-26-01297-s001.zip › figure S5.tif]

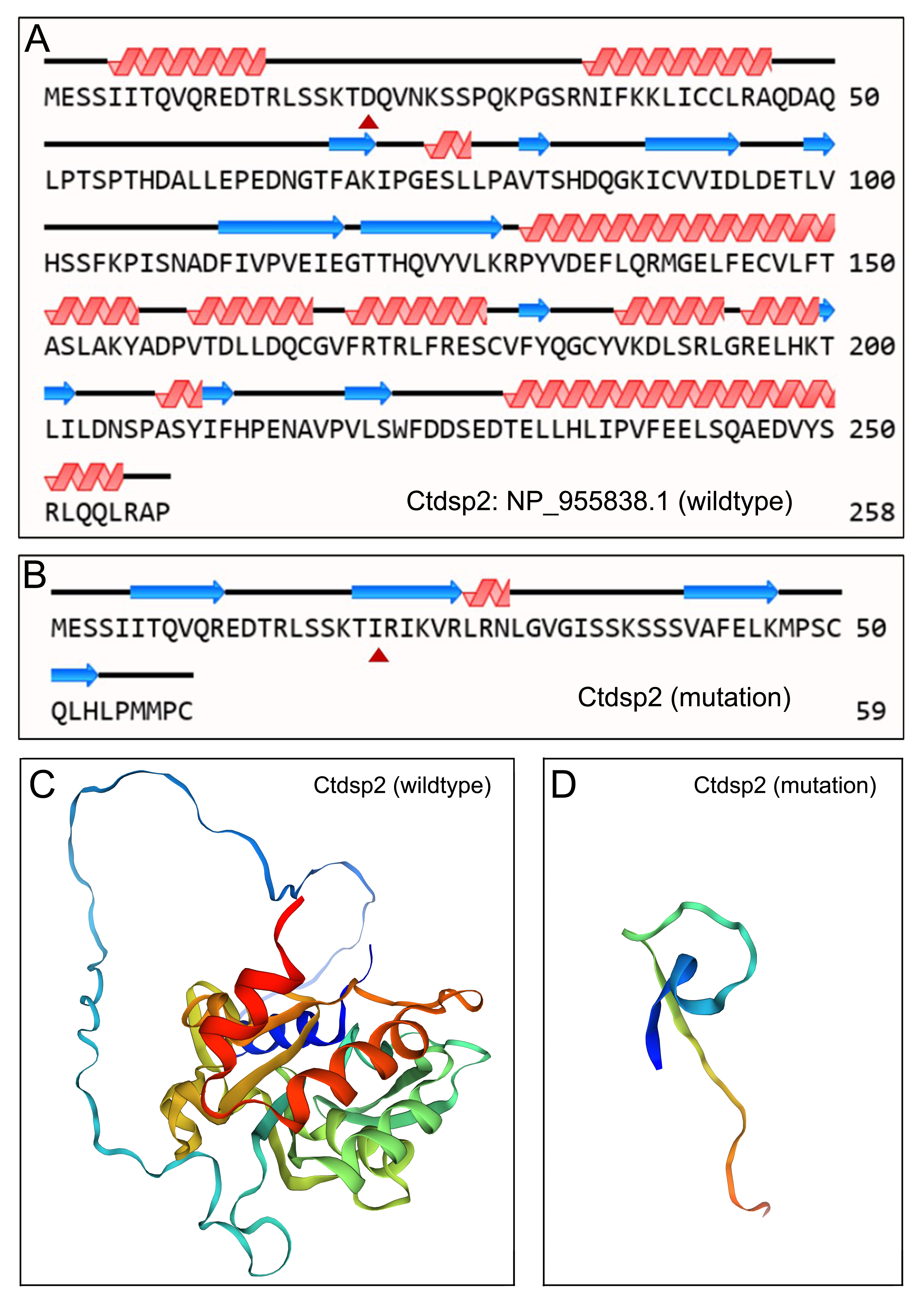

Supplement: Supplementary file 1 [file ijms-26-01297-s001.zip › figure S1.tif]

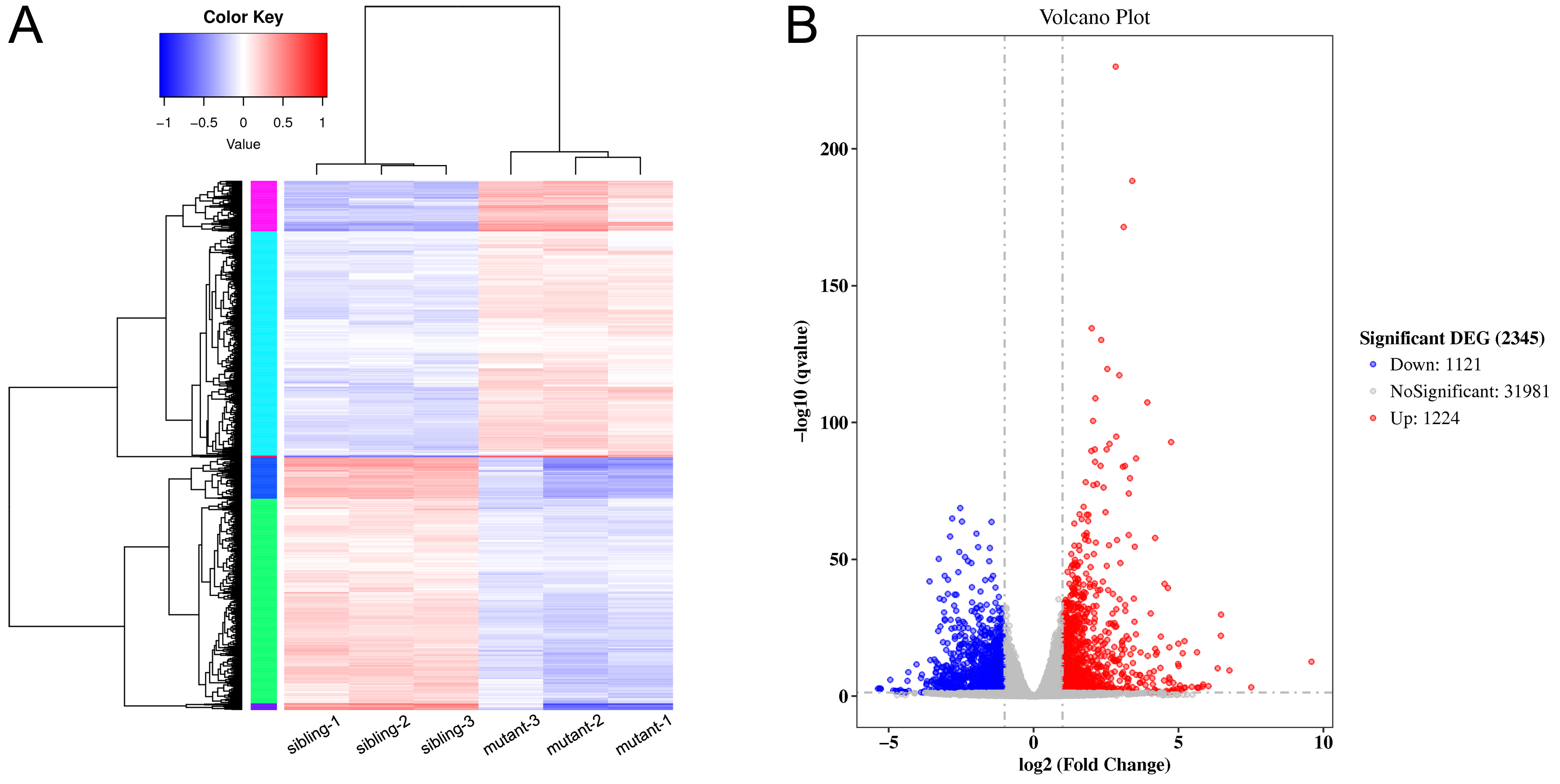

Supplement: Supplementary file 1 [file ijms-26-01297-s001.zip › figure S3.tif]
